# Boredom Proneness and Rule-Breaking: A Persistent Relation One Year into the COVID-19 Pandemic

**DOI:** 10.3390/bs12080251

**Published:** 2022-07-24

**Authors:** Allison C. Drody, Lydia J. Hicks, James Danckert

**Affiliations:** 1 Department of Psychology, University of Waterloo, 200 University Avenue West, Waterloo, ON N2L 3G1, Canada; jdancker@uwaterloo.ca; 2 Department of Psychology, Lakehead University at Thunder Bay, 955 Oliver Rd, Thunder Bay, ON P7B 5E1, Canada; lhicks2@lakeheadu.ca

**Keywords:** boredom proneness, rule-breaking, COVID-19, self-control, pandemic

## Abstract

Research conducted within the first year of the pandemic demonstrated that boredom prone individuals were more likely to break rules (e.g., social distancing) aimed at preventing the spread of COVID-19. It is of interest whether this relation persisted deeper into the pandemic, given that initial results may have reflected the extraordinary nature of the early stages of the pandemic on one hand, or more stable dispositions on the other. Therefore, in the Summer of 2021, we administered an online survey to investigate whether boredom proneness predicted COVID-19 rule-breaking over one year into the pandemic (and approximately one year after the earlier studies). We found that boredom prone individuals remained more likely to engage in COVID-19 rule-breaking. Our results suggest that a trait disposition towards boredom exerts a persistent, long-term influence on behaviour, one that is detrimental to personal well-being during the pandemic. Adherence to public health measures might be improved by encouraging individuals to find adaptive ways of coping with boredom.

## 1. Introduction

During the early stages of the SARS-CoV-2 (COVID-19) pandemic, several countries implemented measures aimed at reducing the spread of the virus. In most instances, this entailed moving work and school-related activities online and encouraging individuals to stay home and maintain social distancing. These measures were not completely adhered to by many individuals [1,2], however, which raised questions about why people would choose not to comply with measures intended to protect their own health and the health of others.

Some have addressed this question by exploring how certain traits and preferences predict adherence to COVID-19 prevention measures. Factors that have been found to be negatively associated with adherence to these measures include a high degree of focus on short-term outcomes and a relative lack of focus on long-term outcomes [3,4], conservative political beliefs [3,5], and low trust in the government or greater trust in social media as resources for obtaining information about COVID-19 [6]. Another important factor that may explain low adherence to these pandemic-related measures is boredom.

Boredom has been described as the negative state that occurs when people want to engage their attention in an activity but are unable to do so [7,8]. The theorized function of this state is to signal when opportunity costs (i.e., the costs of engaging in an activity at the expense of not being able to complete others) are high and to encourage the pursuit of other novel activities [9,10]. Whether or not one responded to instances of boredom in ways that complied with COVID-19 rules might have depended in part on individual differences in boredom proneness, a trait characterized by a tendency to frequently and intensely experience boredom [11]. Given these characteristics, it is possible that boredom prone individuals struggled more than most with feelings of boredom during the pandemic. Moreover, there is reason to believe that these individuals might have had more difficulty resisting the temptation to break COVID-19 rules. Specifically, boredom prone individuals commonly score low on measures of self-control [12,13] while scoring high on traits such as impulsivity [14] and sensation-seeking [15]. Consistent with this explanation, findings from numerous studies suggest that boredom prone individuals may be more likely to cope with boredom in ways that demonstrate a failure to effectively regulate their behaviours. For instance, these individuals have been shown to be more likely to engage in disruptive behaviours at work [16], problematic smartphone use [17,18] and even pathological gambling [19]. On the topic of rule-breaking, studies conducted within the first year of the pandemic demonstrated that high boredom proneness and low self-control were predictive of increased pandemic rule-breaking [20,21], with one study finding that boredom proneness predicted pandemic rule-breaking even when controlling for self-control [20].

It is unclear, however, whether the relation between boredom proneness and COVID-19 rule-breaking would persist in the later stages of the pandemic. It is possible, for instance, that this relation resulted from the abrupt changes individuals faced early in the pandemic and faded as the pandemic progressed. In line with this view, research conducted within the first year of the pandemic showed that experiences of state boredom decreased over time [22], raising the possibility that individuals may have adapted to the pandemic and become more adept at finding solutions to boredom. However, if individual differences in a stable trait (i.e., boredom proneness) reliably give rise to rule-breaking, it seems likely that the relation between boredom proneness and rule-breaking would hold throughout the duration of the pandemic. It is also worth noting that, rather than adapting to the pandemic, some could have grown more impatient and thus more willing to break the rules over time due to factors such as the length of the pandemic and oscillations in implementation of COVID-19 prevention measures.

In our current study, we administered an online survey study in the Summer of 2021 to explore whether boredom proneness predicted pandemic rule-breaking one year into the pandemic (and approximately one year after the original findings [20,21]).

## 2. Materials and Methods

Before data preprocessing, our sample consisted of 448 participants recruited through Amazon Mechanical Turk. Based on the 417 complete responses to our demographic questions, the age range of our sample was 20 to 78 years old (*M_age_* = 39.16, *SD_age_* = 11.05) and our sample consisted of 272 men, 144 women, and 1 individual who preferred to self-identify. Our study was completed by participants from Canada and the United States, and participants received $7.50 USD in exchange for taking part in the study. To remain consistent with previous research on boredom proneness and rule-breaking [20], individuals were required to have a 95% approval rating and a minimum of 500 HITs to participate.

Participants completed a large survey study on Amazon Mechanical Turk that consisted of a variety of self-report measures. Here, we report only the measures and results pertaining to our current research question; however, the full survey, along with the data for this study, are provided as supplementary materials on the Open Science Framework (OSF; https://osf.io/wbqx7/, accessed on 15 July 2022). Participants accessed the survey through a link provided on Mechanical Turk. At the start of the survey, participants were asked to complete three open-ended, text entry questions (taken from [20]) intended to identify potential bots and non-serious responders. The first question asked, “which number is 20 percent of 400?”, the second asked participants to type the phrase “bot not am I” backwards, and the third asked participants to explain whether government-issued ID should be required to vote in local and national elections. Nonsense responses to this last question (e.g., “I agree”) were taken as indications of non-human responders.

Prior to analyzing the data for the current study, we inspected responses to our bot check questions. No answers to these questions suggested that responses were provided by bots or non-serious responders; however, we removed 27 participants who failed to respond to any of the three bot check questions. We also removed 29 participants who attempted to complete the survey more than once, as well as 10 participants who failed to provide responses on the measures of interest in this study. Finally, mahalanobis distance was used to detect multivariate outliers in our regression analyses. This resulted in the removal of six participants. After data preprocessing, our sample consisted of 376 participants (246 male and 130 female), with an age range of 20 to 78 years old (M*_age_* = 38.90, SD*_age_* = 10.87). The ethnic breakdown of our sample can be found in Table 1.

We assessed trait-level self-control using the Brief Self-Control Scale [23]. The Brief Self-Control Scale consists of 13 items, nine of which are reversed scored. Participants are asked to rate their agreement with these items on a Likert scale ranging from 1 (not at all like me) to 5 (very much like me). Questions include “I am good at resisting temptation” and “I wish I had more self-discipline” (reverse-scored). Scores on this scale are summed, with higher scores indicating higher levels of self-control. Tagney and colleagues [23] report internal consistencies of 0.83 and 0.85 for this measure.

To measure trait boredom proneness, we employed the Short Boredom Proneness Scale [24]. The Short Boredom Proneness Scale requires participants to rate their agreement with eight questions, including “I find it hard to entertain myself” and “Much of the time, I just sit around doing nothing”. Responses are provided using a Likert scale ranging from 1 (strongly disagree) to 7 (strongly agree) and scores are calculated by summing responses to each of the items. Higher scores on the scale correspond to higher levels of boredom proneness. Struk and colleagues [24] report an internal consistency of 0.88.

The eight questions used to evaluate COVID-19 rule-breaking were taken from Boylan et al. [20] and are presented in Table 2. As in Boylan et al. [20], to create a composite score of pandemic rule-breaking that could be used in our regression analysis, we employed principal components analysis, which indicated a single latent factor accounting for 31.70% of the variance across four of the eight rule-breaking questions. These results differ from those of Boylan et al. [20], whose rule-breaking factor consisted of six items that together accounted for approximately 55% of the variance. This discrepancy may stem from several factors, such as differences in sample sizes, the time that had elapsed since the initial work on boredom proneness and pandemic rule-breaking was conducted [20,21], and the fact that our rule-breaking questionnaire consisted of eight questions as opposed to nine. Regardless, given that principal components analysis (a commonly used data reduction method [25]) was used to create the rule-breaking factor based on participants’ responses to the rule-breaking items in the present, well-powered study, our analyses continue to provide useful insight into the relation between boredom proneness and rule-breaking.

Factor loadings for items assessing pandemic rule-breaking can be found in Table 2. Participants’ frequency of going out for in-person social visits, shopping in-person and having at-home social gatherings positively loaded onto the rule-breaking factor, whereas spending time in one’s household negatively loaded onto this factor. Higher scores on the rule-breaking factor corresponded to less adherence to COVID-19 prevention measures.

## 3. Results

### 3.1. Bivariate Correlations

We first examined bivariate correlations between our measures of interest (Table 3). Age and self-control were negatively associated with COVID-19 rule-breaking, while boredom proneness was positively associated with these behaviours. Since gender was a categorical (binary, as participants only selected the “male” or “female” options) variable, we employed a series of independent-samples *t*-tests to determine whether boredom proneness or self-control differed significantly as a function of gender. Neither boredom proneness, t(374) = 1.20, *p* = 0.231, nor self-control, t(374) = 0.03, *p* = 0.978, differed between genders.

### 3.2. Hierarchical Regression

To determine whether boredom proneness significantly predicted COVID-19 rule-breaking when controlling for theoretically important variables (i.e., age, gender and self-control), we conducted a hierarchical multiple regression (Table 4). Age was the only predictor in the first step of the model, and it significantly negatively predicted rule-breaking, accounting for 2.38% of the variance in the model. Gender was added in the second step of the model; however, it did not emerge as a significant predictor of rule-breaking, nor did it contribute any additional explained variance to the model. Boredom proneness was added in the third step of the model and was a significant predictor of rule-breaking, explaining an additional 23% of variance in our model. Finally, self-control was added in the fourth step; however, this variable did not significantly improve the model.

## 4. Discussion

Research conducted within the early stages (i.e., within the first few months) of an extraordinary world event—the COVID-19 pandemic—revealed that those high in boredom proneness were more likely to break rules aimed at preventing the spread of COVID-19 [20,21]. The present work was conducted to determine whether the relations observed would hold true, one year later. Consistent with prior research [20,21], we found that boredom proneness predicted rule-breaking, even when controlling for theoretically relevant variables, such as trait-level self-control. In fact, self-control explained no additional variance when added in the final step of our model. Although there is some evidence to suggest that individuals may have adapted to the pandemic, experiencing less boredom over time [22], our results suggest that those higher in trait boredom proneness continue to be more likely to act in ways that involve disobeying pandemic-related regulations, potentially risking their own health and the health of others. Why might boredom prone individuals be more likely to break rules? A likely explanation comes from our understanding of boredom prone individuals, as well as current accounts of state boredom.

Individuals high in trait boredom proneness report frequently and intensely experiencing the state of boredom [11], a state that has been theorized to encourage individuals to pursue new activities when opportunity costs are high [9,10]. This account of state boredom was tested by Struk and colleagues [10]. In their experiment, participants were placed in one of two rooms and informed that they would only be allowed to engage with their own thoughts throughout the duration of the experiment. One of the rooms was empty, whereas the other room contained several possible activities (e.g., puzzles and drawing materials). Importantly, in this room participants were told they were not to engage with any of the obviously available options for action. As such, this room was considered higher in opportunity costs than the empty room. The researchers found that boredom was higher in the room with options for engagement that participants were prevented from engaging with when compared to the empty room, providing support for the notion that conditions of high opportunity costs give rise to boredom. One can easily draw parallels between this experiment [10] and the conditions imposed on individuals following the implementation of pandemic-related restrictions. That is, during the pandemic, people had opportunities to take part in numerous everyday activities, such as holding indoor gatherings or shopping for leisure, but were asked to refrain from engaging in these activities. It is therefore unsurprising that the number of internet searches related to boredom [26], as well as reports of feeling bored [22], increased shortly after these measures took effect. Importantly, these increases in boredom during the pandemic might have been felt most strongly by boredom prone individuals [11].

It should be noted that, although feelings of state boredom are thought to encourage the pursuit of novel tasks [9,10], they do not guarantee that individuals will choose adaptive solutions to boredom. Therefore, individuals could have responded to pandemic boredom in ways that complied with COVID-19 regulations (e.g., engaging in solitary hobbies) or in ways that involved breaking the rules (e.g., gathering in large groups). Considering that boredom prone individuals tend to present with poor self-control [12,13] as well as high sensation-seeking [15] and impulsivity [14], it seems possible that they may have had more difficulty resisting the temptation to engage in rule-breaking when searching for ways to alleviate boredom. 

Our findings provide insight into some ways in which governments and institutions might encourage compliance to public health measures as the pandemic continues or should similar incidents arise in the future. One approach might entail communicating adaptive ways of coping with boredom during the pandemic, perhaps by highlighting activities in which one could engage while still complying with pandemic-related regulations. In addition, preemptively creating a plan for dealing with one’s boredom in positive ways has been shown to alleviate boredom [27]. These strategies might improve adherence by helping boredom prone individuals to find ways of overcoming boredom that do not involve breaking pandemic-related regulations and should be shared with the public whenever these types of regulations are being implemented. This information could be disseminated in a variety of ways, such as through public advertisements or COVID-19 information sites.

While our findings carry important implications for reducing COVID-19 rule-breaking, we acknowledge that our study is not without limitations. One potential concern regarding our study design is that it does not enable us to make any strong causal claims about the relation between boredom proneness and COVID-19 rule-breaking. However, since boredom proneness is a relatively stable trait [28,29] and individuals have been living through a pandemic for only two years, it seems reasonable to assume that individual differences in this trait have some effect on individuals’ rule-breaking behaviours. Another limitation of our work is that our sample was restricted to Mechanical Turk participants recruited from Canada and the United States. It should be noted that pandemic-related measures as well as adherences to these measures have been shown to vary across countries and cultures [30,31]. Moreover, there is evidence to suggest that cultures may differ in their methods of coping with boredom [32,33]. It is therefore unclear to what extent our results would generalize to broader samples. Future research could explore whether boredom proneness poses similar threats to compliance with pandemic-related regulations beyond North America using more diverse samples.

Another potential limitation regarding the generalizability of our findings concerns the fact that this study was conducted in the Summer of 2021. Currently (in the Summer of 2022), many countries have reduced or lifted most pandemic-related restrictions, and it remains unknown whether boredom prone individuals would still be more likely to engage in COVID-19 rule-breaking having lived through two years of a global pandemic. There is reason to believe that the relation between boredom proneness and rule-breaking would hold in countries that continue to enforce pandemic-related restrictions, considering that these types of restrictions should foster conditions of high opportunity costs and increased feelings of boredom. Moreover, the relation between boredom proneness and COVID-19 rule-breaking appears to be stable, having persisted over the course of at least one year of the pandemic. In regions that have loosened or abolished restrictions, certain health-promoting behaviours, such as mask wearing and social distancing, are still encouraged. An interesting avenue of research could therefore involve exploring whether individual differences in boredom proneness predict tendencies to engage in health-promoting behaviours more broadly. Another potentially useful question for future research concerns how boredom prone individuals coped during the pandemic when they were not engaging in rule-breaking. Maladaptive behaviours, such as problematic smartphone use [17,18], substance use [34,35] and pathological gambling [19], are prevalent among those high in boredom proneness; however, it is unknown whether some of these behaviours have increased among these individuals over the course of the pandemic. Exploring these questions could further illuminate the ways in which governments and institutions can promote the health of citizens throughout the remainder of the pandemic.

To conclude, our investigation of the relation between boredom proneness and COVID-19 rule-breaking revealed that, over one year into the pandemic, boredom prone individuals remained more likely to engage in pandemic rule-breaking. Our results suggest that this trait exerts persistent and long-term influences on behaviours that can threaten one’s own health as well as the health of others. Governments and institutions attempting to improve adherence to COVID-19 regulations should consider communicating to the public adaptive ways of coping with boredom while restrictions are in place.

## Figures and Tables

**Table 1:** Participants’ self-reported ethnicities.

Ethnicity	Frequency	Percentage
Caucasian	304	80.85
First Nations (North American Indian)	5	1.33
Métis	1	0.27
Other European Origins	1	0.27
Black or African American	31	8.24
Carribbean Origins	2	0.53
Latin, Central or South American Origins	12	3.19
West Central Asian and Middle Eastern Origins	3	0.80
South Asian Origins	14	3.72
East and South Eastern Origins	20	5.32
Other Asian Origins	2	0.53
Oceania Origins	1	0.27
Other/Prefer to Self-Identify	3	0.80

Note. Participants could select multiple categories to indicate which race(s) they identified with. This table presents the number of participants who endorsed each option. Therefore, the sum of the numbers presented in this table will not correspond to 100%. *N* = 376.

**Table 2:** Rule-breaking questions and accompanying factor loadings.

Rule-Breaking Questions	Response Options	Factor Loadings
On average, how many hours of the day are you spending in your household (including your garage or yard but not going into the neighbourhood or other public spaces)?	1–24	−0.79
How frequently have you gone out for in-person social visits?	1 (not at all)–5 (constantly)	0.86
How frequently do you go out to shop in-person?	1 (not at all)–5 (constantly)	0.78
In the past week, how many social gatherings have you had at your home (i.e., gatherings with people other than those with whom you live)?	Free numerical entry	0.57
Not included in rule-breaking factor		Correlation with factor
To what extent are you practicing social distancing over the past week?	1 (not at all)–4 (very much)	−0.16 **
How many people have come within 6ft of you over the last week (best guess, other than people who live with you in your household)?	Free numerical entry	−0.08
How many days have you spent in isolation over the past week?	1–7	−0.19 ***
To what extent are you washing your hands with soap and water in response to the COVID-19 pandemic?	1 (not at all)–4 (very much)	0.03

Note. Principal components analysis was conducted with *N* = 382 since outlier removal based on mahalanobis distances had to be conducted after the rule-breaking composite score was calculated for each participant. ** *p* < 0.01, *** *p* < 0.001.

**Table 3:** Bivariate correlations involving age, self-control, boredom proneness and COVID-19 rule-breaking.

	Age	Self- Control	Boredom Proneness
Self-Control	0.18 ***		
Boredom Proneness	−0.29 ***	−0.71 ***	
COVID-19 Rule-Breaking	−0.15 **	−0.34 ***	0.51 ***

Note. ** *p* < 0.01, *** *p* < 0.001. *N* = 376.

**Table 4:** Hierarchical regression predicting COVID-19 rule-breaking.

	*b*	*SE*	*B*	*t*	*p*	*R^2^∆*	*P∆*
Step 1							
Age	−0.01	0.00	−0.15	−3.02	0.003		
						0.02	0.003
Step 2							
Age	−0.02	0.00	−0.18	−3.36	<0.001		
Gender	0.19	0.10	0.10	1.87	0.063		
						0.01	0.063
Step 3							
Age	−0.00	0.00	−0.03	−0.61	0.544		
Gender	0.19	0.09	0.10	2.13	0.034		
BPS	0.04	0.00	0.51	10.91	<0.001		
						0.23	<0.001
Step 4							
Age	−0.00	0.00	−0.03	−0.59	0.553		
Gender	0.20	0.09	0.10	2.16	0.031		
BPS	0.04	0.00	0.54	8.28	<0.001		
BSCS	0.00	0.01	0.04	0.71	0.480		
						0.00	0.480

*N* = 376.

## Data Availability

Data for the present study is publicly available on the OSF at the following link: https://osf.io/wbqx7/ (accessed on 15 July 2022).
